# Prevalence and genotype distribution of potential high-risk and high-risk human papillomavirus among women attending selected reproductive health clinics in lake victoria basin-kenya: a cross-sectional study

**DOI:** 10.1186/s12905-024-03303-9

**Published:** 2024-08-24

**Authors:** Ivy Akinyi, Shehu Shagari Awandu, Davy Vanden Broeck, Ana Rita Pereira, Nina Redzic, Johannes Bogers

**Affiliations:** 1 School of Health Sciences, Jaramogi Oginga Odinga University of Science and Technology, Bondo, Kenya; 2 AML, Sonic Healthcare, Antwerp, Belgium; 3 Laboratory of cell biology and histology, University of Antwerp, Antwerp, Belgium; 4 International Centre for Reproductive Health, Ghent University, Ghent, Belgium

**Keywords:** Human papillomavirus, Cervical cancer, Genotype, Kenya

## Abstract

**Background:**

Persistent human papillomavirus (HPV) infection is considered the primary etiological factor for invasive cervical cancer. Understanding the epidemiology of circulating potential high-risk (HR) and HR HPV strains is essential in updating epidemiological knowledge and recommendations on genotype-specific vaccination development. This study determined the prevalence and factors associated with Potential HR/HR HPV among women attending selected reproductive health clinics in Lake Victoria Basin.

**Methods:**

A cross-sectional facility-based survey made up of 434 women aged 16–68 years was carried out in two selected facilities. Structured questionnaires were administered to collect participant clinical and social characteristics. Cervical specimens were collected and HPV genotyping was carried out using RIATOL HPV genotyping qPCR assay. Descriptive statistics followed by logistic binary regression was done using R version 4.3.2.

**Results:**

The overall prevalence of potential HR/HR HPV among women attending the selected reproductive health clinics was reported at 36.5% (158/434). Specifically, in the rural setting, Gobei Health Center, the prevalence was 41.4% (41/99) while in the urban setting-JOOTRH, it was 34.6% (117/335). The most prevalent potential HR/HR HPV are 52, 67, 16, 31, 39, 45, and 31 among women. Age was the main factor associated with HPV infection with women between the age of 30–39 having the highest risk (AOR = 0.3, CI:0.2–0.7, *p* < 0.001).

**Conclusion:**

In both rural and urban regions, potential HR/HR HPV infection among women attending reproductive health clinics at the selected facilities remains common. The study identifies the need for effective implementation and clinical follow-up process of cervical cancer control program in the LVB.

**Supplementary Information:**

The online version contains supplementary material available at 10.1186/s12905-024-03303-9.

## Background

According to estimates, cervical cancer is the fourth most frequent cancer in women with up to 604,000 new cases globally in 2020 [1]. Primarily, cervical cancer is regarded as the major cause of cancer-related deaths among women worldwide. Of the estimated 342,000 deaths that occurred in 2020 due to cervical cancer, 90% of these cases were found in low and middle-income countries [2–4]. The world’s largest cervical cancer burden is reported in the Sub-Saharan Africa (SSA). Cervical cancer mortality and incidence have grown in Kenya. Annually, it is estimated that there are up to 5236 new cases and about 3211 deaths occur among women in Kenya between 15 and 44 years [5]. This is attributed to low cervical cancer screening uptake, insubstantial treatment and care, and lack of HPV vaccine among women [6].WHO in 2020, launched a global cervical cancer elimination strategy, identifying key interventions (also referred to as the 90; 70; 90 cascades) to be implemented by various nations across the globe by 2030 [7]. It projects that by 2030, 90% of girls should be fully vaccinated against HPV by the age of 15; 70% of women screened with a high precision test at least twice between age 30 and 49 years; 90% of women with pre-cancer and invasive cervical cancer should receive care and treatment as a pathway to eliminate cervical cancer [7].

Previous studies attribute the surging cases of cervical cancer to the rising and persistent cases of Human papillomavirus (HPV) (also believed to be the etiological factor) [8]. There are more than 200 HPV genotypes that have been identified. However, about 40 HPV genotypes been established to infect the mucosal epithelium with includes the anogenital epithelium [9]. HPV infection have two possible competing outcomes: clearance or progression; Up to 90% of HPV infections clear naturally. When infection fails to clear, it is said to be persistent and progresses to precancerous and cancerous lesions [10]. HPV can be classified as being High Risk (HR), potential HR, or Low Risk (LR) based on the genotype and oncological risk. For instance, cervical cancer is caused by High-Risk-HPV [11] including HPV 16, 18, 31, 33, 35, 39, 45, 51, 52. The potential HR include 26, 53, 56, 66, 67,68, 73, 82. The LR HPV include 6, 11, 40, 42, 43, 44, 54, 61, 70, 72, 81. HPV 16 and HPV 18 account for 70% of cervical cancer cases globally [11]. It is well-known that the oncogenicity of the HPV genotypes is largely increased by factors such as Human Immunodeficiency Virus (HIV) infection, multiple sex partners, age of first sexual debut, smoking, use of alcohol, and parity among others [12].

Furthermore, the United Nations’ Sustainable Development (SDG) Goal 3 was adopted as part of the agenda 2030 to ensure healthy lives and wellbeing for all [13]. One of its specific targets is to lower mortality attributed to non-communicable diseases (NCDs) such as cervical cancer through prevention and treatment [14]. In developed nations, a significant reduction in cervical cancer cases is attributed to proper implementation of WHO guidelines which include timely screening of sexually active women, HPV vaccination of girls from the age of 10 years, and comprehensive treatment of cervical cancer patients [7]. The three approved prophylactic HPV vaccines by the US food and Drug Administration for vaccination are Gardasil-4 (a tetravalent vaccine for HPV type 6,11,16,18), Cervarix (a bivalent vaccine for HPV 16 and 18), and a Gardasil-9, a nona-valent vaccine targeting nine HPV genotypes (6,11,16,18,31,33,45,52,58) [15]. However, in developing countries such as Kenya, the trend in cervical cancer cases has remained upward posing a challenge to achievement of the SDG 3 despite the administration of either of the three HPV vaccines. Further, the uptake of cervical cancer screening in Kenya has been low standing at 16.8% in 2022 [16]. There are three main screening approaches for cervical screening in Kenya. HPV testing is considered the primary screening strategy for women above the age of 30 years. HPV DNA detection methods often used include use of polymerase Chain Reaction (PCR), Southern Blot Hybridization, In Situ Hybridization among others. The second strategy is the use of Visual Inspection with Acetic Acid (VIA) or Visual Inspection with Lugol’s (VILI) which is the most commonly used in many primary facilities because of its cost effectiveness. The third method is the Pap smear which is mainly used among women who are not eligible for VIA/VILI [17].

Previous studies conducted in Kenya have found a significant prevalence of HR HPV especially in the Urban regions [18, 19]. However, in the HIV-burdened Lake Victoria Basin, prevalence studies are scanty. Also, epidemiological knowledge on potential HR HPV types is extremely limited, mainly because most commercial molecular assays can detect HR HPV only. Further, due to population increase, urbanization, and the recent COVID-19 pandemic, there may have been changes over time in both the prevalence and synergetic factors associated with potential HR and HR HPV infection and therefore disease occurrence. Given that, it is of utmost importance to generate updated epidemiological data on potential HR and HR HPV among women visiting selected reproductive health Clinics in LVB-Kenya.

## Methodology

### Study design, area and, population

A facility-based cross-sectional study was undertaken at the two selected reproductive health clinics; Jaramogi Oginga Odinga Teaching and Referral Hospital-JOOTRH (Kisumu County) and Gobei Health Center (Siaya County) between July and September of 2023. The reproductive health clinics are known to offer standard services such as family planning services, reproductive health counselling, gynecology consultations and cervical cancer screening. The inclusion criteria included women who presented themselves to the clinic for any gynecological reason, including cervical cancer screening, and were aged between 16 and 68 years. The exclusion criteria involved women who had undergone total hysterectomy, women with visible cervical lesions and women with immediate bleeding on contact.

**Fig. 1:**
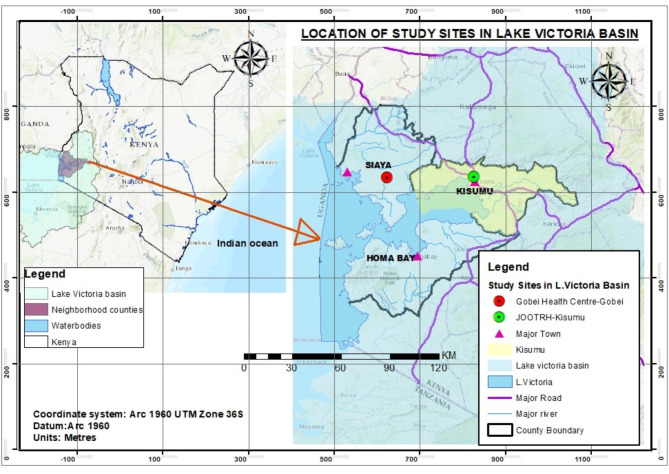
Map of Kenya showing the selected study sites

A non-probability convenience sampling technique was utilized to select the two study sites based on location (Fig. 1), scope, and volume of services offered. Gobei health center is located in the rural setting of Siaya County and draws its population from the inhabitants who are mainly peasant farmers and small-scale traders of farm produce. The facility has limited infrastructure, an inadequate number of staff, and inconsistencies in cervical cancer screening in the area owing to frequent run out of consumables. In contrast, JOOTRH is an established referral facility based in the capital city of Kisumu County (Kisumu Town). The town is a metropolitan city with a mixed urban population drawn from different parts of the massive Lake Victoria Basin. It is known for consistent services of cervical screening programs due to the availability of patients from the town itself and other possible referrals.

### Sample size determination

The sample size was calculated using the Kish Leslie formula (1965), where n refers to the estimated minimum sample size required, p is the prevalence of a characteristic in a sample, e is the acceptable margin of error (5%), and z the confidence interval (CI) set at 1.96 for a 95% CI.

Given the population-based cross-sectional study by Omire et al. (2020) among HIV-positive and negative participants at a reproductive health clinic in Nairobi, Kenya [20] the prevalence of HPV in Kenya was set to 31.3%, therefore setting the n to 329. Accounting for a 10% non-response rate (33 participants), the final sample size required for this study was calculated to be 362.

All women who visited these facilities during the study period and were eligible for the study were consecutively enrolled until the number of participants reached the minimum sample size calculated; A total of 335 women were enrolled in JOOTRH and 99 in Gobei Health Center. The initial calculated sample size was 362 (275-JOOTRH and 87 Gobei Health Center) was allotted to the two facilities on the basis of volume and scope of coverage. However, participants continued to be recruited on their request until the new sample size of 434. While this figure was not anticipated, the researcher gave room for reasonable enrollment as it would still improve precision and bring out any outliers.

### Sample collection/storage

After obtaining informed consent from the study participants, each participant was given standardized questionnaires in English or local languages (Luo) to fill and those who needed assistance were helped by the research assistants. The structured questionnaires focused on their socio-demographic characteristics, past reproductive health history, sexual behaviors, and other risk factors associated with potential HR/HR HPV (see supplementary information). The HIV status of each woman was confirmed from the hospital data registry where each woman receiving reproductive health services is encouraged to undergo HIV testing before getting routine services and their results are recorded in Ministry of Health registry for HIV Surveillance. Each woman consented to have this information obtained for the purpose of this study only. To maintain privacy, a registered Reproductive Health Clinic nurse conducted a physical examination and sample collection in a separate room. A sterile vaginal speculum was inserted into the vagina to obtain a cervical swab for HPV testing. The specimens were obtained using the multi-collect specimen collection kit (Abbott). The collection swab was rotated twice in the ectocervical and endocervical region and then deposited into 1.2 ml of transport buffer that contains guanidine thiocyanate (for DNA stabilization), according to manufacturer instructions. The specimens were then stored at -80℃ in the Western Kenya Cancer Care and Research Center laboratory for preservation till shipment to Antwerp, Belgium for molecular analysis.

### RIATOL HPV genotyping qPCR assay

The RIATOL qPCR HPV assay was used to extract and genotype HPV-DNA at AML, Sonic Healthcare Benelux (Antwerp, Belgium) under ISO15189 accreditation, as previously described [1]. The choice to use RIATOL HPV genotyping qPCR assay was decided on because of its proven clinical accuracy, reproducibility and quantifiable results as it provides viral load information. Briefly, cervical samples were collected with the multi-Collect Specimen Collection Kit (Abbott) and 300 µl of each sample was transferred into the 96-deep well plate by using Janus G3 (Perkin Elmer) robot for batch processing. DNA was extracted in the automatic nucleic preparation in Chemargic360 (Perkin Elmer) using the viral DNA/RNA 360 H96 extraction kit. The DNA extract was then stored at 4℃ awaiting PCR. As an internal cellular control, beta-globin was employed to assess the validity of the PCR run. To achieve relative quantification of data and confirm the qPCR acceptable efficiency ranges of 90–110%, standard curves were generated by amplification of a series of synthetic DNA ten-fold dilutions. Subsequently, 384-PCR well plates were prepared by the Janus Liquid handler robot (Perkin Elmer) by mixing 2 µL of extracted DNA or specific control with 4 µL of the assigned master mix solutions, as described [1]. In total, each sample was tested with 8 different master mixes, including primers and probes for detection of HPV 6, 11, 16, 18, 31, 33,35, 39, 45,51, 52, 53 56, 58, 59, 66, 67, 68 and beta-globin. RT-amplification was carried out on the Light Cycler 480 (Roche) instrument. The thermo-cycling profile consisted of 45 two-step cycles: 10s at 95℃ and 30s at 60℃. At the end of the amplification run, the RT-PCR run file automatically was transferred to the Fast-Finder online AI platform (Velsera) for analysis where clinical cut-offs were applied to determine positive and negative samples. A detailed description of the assay design and validation is available in Micallesi et al., [1].

### Data management and analysis

Data were entered by data clerks into Excel sheets twice. Using R version 4.3.2 data validation, cleaning, and analysis were performed. Prevalence of potential HR/HR HPV was calculated as a proportion of study participants expressed as a frequency and percentage. The variables were then explored using tabulations. Specifically, the factors associated with potential HR/HR HPV were analyzed using logistic regression (bivariate and multivariate level). Variables that showed statistical significance at the bivariate level or those that had *p* < 0.2 were specifically transferred to the multivariate level. Statistical significance was considered at p-value ≤ 0.05.

## Results

### Socio-demographic, reproductive health history, and past sexual behavior characteristics

#### Rural-gobei health centre

The median age of women attending the Gobei of Health center-rural setting was 30 [24, 36]. Majority of the women were between the age of 30–39 years (29.3%, 29/99). Majority of women confirmed that this was their first time to be screened-initial screening (69.7%, 69/99), and were HIV positive (47.5%, 47/99). In addition, more than three-quarters of the women were married (76.8%, 76/99). The highest level of education was primary (53.5%, 53/99). More than half of the women were unemployed (54.5%, 54/99). Furthermore, majority of the women had two/three children (41.4%, 41/99), almost two thirds used both hormonal and non-hormonal contraceptives (62, 62/99), and majority age of first sex was between 15 and 19 years (70.7%, 70/99) (See Table 1).

### Urban-JOOTRH

The median age of women attending the JOOTRH-Urban setting was 33(27,40). Majority of the women were between the age of 30–39(39.1%, 131/335). Unlike in the rural setting, more than half of women confirmed having undergone previous Screening-Routine screening (55.5%, 186/335) and were HIV positive (56.7%, 190/335), In addition, two-thirds of the women were married (65.4%, 219/335). Unlike in rural Gobei Health Centre, the highest level of education was high-school education (36.1%, 121/335) and more than half were self-employed/Business (59.4%, 199/335). Further, the majority of the women had two/three children (44.2%, 148/335), used contraceptives (79.4%, 248/335), and the age at first sex was 15–19 years (64.5%, 216/335) (See Table 1).

### Prevalence of potential HR/HR HPV infection-rural(Gobei) and urban(JOOTRH)

The prevalence of potential HR/HR HPV among women attending Gobei Health center was 41.4%(41/99). The most common genotypes of potential HR/HR HPV circulating were 67, 52, 16, 35, 31, 18 in descending frequency (Table 2).

The prevalence of Potential HR/HR HPV among women attending JOOTRH was 34.9%(117/335). The most common genotypes of potential HR/HR HPV include 52, 16, 39, 67, 58, 45, and 31 in decreasing frequency (Table 2).

Overall, in both facilities, the prevalence of potential HR/HR HPV was 36.4%. The potential HR/ HR HPV that were most prevalent include HPV 52 (14.8%), HPV 16 (10.8%), HPV 67(9.7%), HPV 39 (9.1%), HPV 31 (7.4%), and HPV 45 (7.4%).

**Table 1:** The results of bivariate analysis for sociodemographic and Reproductive health history and past sexual history

	Overall, Kisumu	Kisumu			Overall, Siaya	Siaya		
**Variable**	*N* = 335	**No PH/HR HPV**, *N* = 218	**PH/HR HPV**, *N* = 117	**p-value**	*N* = 99	**No PH/HR HPV**, *N* = 58	**PH/HR HPV**, *N* = 41	**p-value**
**Age**	33 (27, 40)	34 (27, 40)	32 (25, 39)	0.051	30 (24, 37)	33 (28, 40)	25 (23, 33)	**0.001**
**Age**				**0.048**				**0.004**
16–24	52 (15.5%)	25 (11.5%)	27 (23.1%)		27 (27.3%)	8 (13.8%)	19 (46.3%)	
25–29	63 (18.8%)	42 (19.3%)	21 (17.9%)		20 (20.2%)	13 (22.4%)	7 (17.1%)	
30–39	131 (39.1%)	90 (41.3%)	41 (35.0%)		29 (29.3%)	20 (34.5%)	9 (22.0%)	
40+	89 (26.6%)	61 (28.0%)	28 (23.9%)		23 (23.2%)	17 (29.3%)	6 (14.6%)	
**Type of visit**				**0.039**				0.527
Initial screening	149 (44.5%)	88 (40.4%)	61 (52.1%)		69 (69.7%)	39 (67.2%)	30 (73.2%)	
Routine screening	186 (55.5%)	130 (59.6%)	56 (47.9%)		30 (30.3%)	19 (32.8%)	11 (26.8%)	
**Marital Status**				**0.014**				0.119
Single	69 (20.6%)	34 (15.6%)	35 (29.9%)		10 (10.1%)	3 (5.2%)	7 (17.1%)	
Married	219 (65.4%)	153 (70.2%)	66 (56.4%)		76 (76.8%)	47 (81.0%)	29 (70.7%)	
Divorced/Separated	25 (7.5%)	15 (6.9%)	10 (8.5%)		1 (1.0%)	0 (0.0%)	1 (2.4%)	
Widowed	22 (6.6%)	16 (7.3%)	6 (5.1%)		12 (12.1%)	8 (13.8%)	4 (9.8%)	
**Level of education**				0.072				0.081
Primary/None	109 (32.5%)	80 (36.7%)	29 (24.8%)		53 (53.5%)	34 (58.6%)	19 (46.3%)	
Highschool	121 (36.1%)	76 (34.9%)	45 (38.5%)		39 (39.4%)	18 (31.0%)	21 (51.2%)	
College/University	105 (31.3%)	62 (28.4%)	43 (36.8%)		7 (7.1%)	6 (10.3%)	1 (2.4%)	
**Emplyment status**				0.659				0.694
Unemployed	61 (18.2%)	37 (17.0%)	24 (20.5%)		54 (54.5%)	32 (55.2%)	22 (53.7%)	
Employed	75 (22.4%)	48 (22.0%)	27 (23.1%)		7 (7.1%)	3 (5.2%)	4 (9.8%)	
Self-employed/Business	199 (59.4%)	133 (61.0%)	66 (56.4%)		38 (38.4%)	23 (39.7%)	15 (36.6%)	
**Number of children**				0.081				**0.044**
0	42 (12.5%)	20 (9.2%)	22 (18.8%)		1 (1.0%)	1 (1.7%)	0 (0.0%)	
1	69 (20.6%)	45 (20.6%)	24 (20.5%)		20 (20.2%)	7 (12.1%)	13 (31.7%)	
2–3	148 (44.2%)	102 (46.8%)	46 (39.3%)		41 (41.4%)	24 (41.4%)	17 (41.5%)	
4+	76 (22.7%)	51 (23.4%)	25 (21.4%)		37 (37.4%)	26 (44.8%)	11 (26.8%)	
**Contraceptive use**				0.084				**0.025**
No	87 (26.0%)	50 (22.9%)	37 (31.6%)		37 (37.4%)	27 (46.6%)	10 (24.4%)	
Yes	248 (74.0%)	168 (77.1%)	80 (68.4%)		62 (62.6%)	31 (53.4%)	31 (75.6%)	
**Had more than one sex partner**				0.380				0.199
No	69 (20.6%)	48 (22.0%)	21 (17.9%)		26 (26.3%)	18 (31.0%)	8 (19.5%)	
Yes	266 (79.4%)	170 (78.0%)	96 (82.1%)		73 (73.7%)	40 (69.0%)	33 (80.5%)	
**Age at first sex**				0.781				0.714
Below 15 years	38 (11.3%)	23 (10.6%)	15 (12.8%)		20 (20.2%)	12 (20.7%)	8 (19.5%)	
15–19 years	216 (64.5%)	143 (65.6%)	73 (62.4%)		70 (70.7%)	42 (72.4%)	28 (68.3%)	
Above 20 years	81 (24.2%)	52 (23.9%)	29 (24.8%)		9 (9.1%)	4 (6.9%)	5 (12.2%)	
**Had STI**				0.331				**0.016**
No	262 (78.2%)	174 (79.8%)	88 (75.2%)		81 (81.8%)	52 (89.7%)	29 (70.7%)	
Yes	73 (21.8%)	44 (20.2%)	29 (24.8%)		18 (18.2%)	6 (10.3%)	12 (29.3%)	
**Had history of cervical cancer in the family**				0.492				0.690
No	320 (95.5%)	207 (95.0%)	113 (96.6%)		93 (93.9%)	55 (94.8%)	38 (92.7%)	
Yes	15 (4.5%)	11 (5.0%)	4 (3.4%)		6 (6.1%)	3 (5.2%)	3 (7.3%)	
**Smokes cigarretes**				0.302				0.646
No	331 (98.8%)	214 (98.2%)	117 (100.0%)		94 (94.9%)	56 (96.6%)	38 (92.7%)	
Yes	4 (1.2%)	4 (1.8%)	0 (0.0%)		5 (5.1%)	2 (3.4%)	3 (7.3%)	
**Ever used alcohal**				0.172				0.157
No	292 (87.2%)	194 (89.0%)	98 (83.8%)		90 (90.9%)	55 (94.8%)	35 (85.4%)	
Yes	43 (12.8%)	24 (11.0%)	19 (16.2%)		9 (9.1%)	3 (5.2%)	6 (14.6%)	
**Number of HPV**				0.572				> 0.999
1	78 (60.5%)	7 (50.0%)	71 (61.7%)		25 (53.2%)	4 (50.0%)	21 (53.8%)	
2–3	43 (33.3%)	6 (42.9%)	37 (32.2%)		15 (31.9%)	3 (37.5%)	12 (30.8%)	
4+	8 (6.2%)	1 (7.1%)	7 (6.1%)		7 (14.9%)	1 (12.5%)	6 (15.4%)	
Missing	206	204	2		52	50	2	
**HIV status**				0.704				0.157
Negative	145 (43.3%)	96 (44.0%)	49 (41.9%)		52 (52.5%)	27 (46.6%)	25 (61.0%)	
Positive	190 (56.7%)	122 (56.0%)	68 (58.1%)		47 (47.5%)	31 (53.4%)	16 (39.0%)	

*Potential High Risk/High Risk HPV-PH/HR HPV

**Table 2:** Distribution of HPV in women attending selected reproductive health clinics

	Women	Site	
**Variable**	*N* = 176	**Kisumu**, *N* = 129	**Siaya**, *N* = 47
**HPV type**			
HPV 11	1 (0.6%)	1 (0.8%)	0 (0.0%)
HPV 16	19 (10.8%)	14 (10.9%)	5 (10.6%)
HPV 18	6 (3.4%)	2 (1.6%)	4 (8.5%)
HPV 31	13 (7.4%)	10 (7.8%)	3 (6.4%)
HPV 33	5 (2.8%)	3 (2.3%)	2 (4.3%)
HPV 35	11 (6.2%)	6 (4.7%)	5 (10.6%)
HPV 39	16 (9.1%)	13 (10.1%)	3 (6.4%)
HPV 45	13 (7.4%)	10 (7.8%)	3 (6.4%)
HPV 51	8 (4.5%)	7 (5.4%)	1 (2.1%)
HPV 52	26 (14.8%)	20 (15.5%)	6 (12.8%)
HPV 53	6 (3.4%)	4 (3.1%)	2 (4.3%)
HPV 56	10 (5.7%)	8 (6.2%)	2 (4.3%)
HPV 58	10 (5.7%)	10 (7.8%)	0 (0.0%)
HPV 59	4 (2.3%)	3 (2.3%)	1 (2.1%)
HPV 6	2 (1.1%)	1 (0.8%)	1 (2.1%)
HPV 66	3 (1.7%)	2 (1.6%)	1 (2.1%)
HPV 67	17 (9.7%)	11 (8.5%)	6 (12.8%)
HPV 68	6 (3.4%)	4 (3.1%)	2 (4.3%)

### Influence of HIV status on prevalence of potential HR/HR-HPV

Type-specific analysis revealed that the most prevalent Potential HR and HR HPV were distinct in both HIV-positive and negative women (See Table 3). In decreasing order, the most prevalent HPV among HIV positive women were HPV 67, 16, 31, 45, 52 while for HIV negative women it was HPV 52, 39, 16, 68, 35 (Table 3). The prevalence of Potential HR/ HR HPV was 53.2% (84/158) among HIV-positive women and 46.8% (74/158) among HIV-negative women. Compared to HIV-negative women, the prevalence of potential HR/HR HPV types was higher in HIV positives 55.1% (97/176).

**Table 3:** Distribution of HPV types among women by their HIV status

Variable	*N* = 176	HIV- *N* = 79	HIV+ *N* = 97
HPV type			
HPV 11	1 (0.06%)	1 (1.3%)	0 (0.0%)
HPV 16	19 (10.8%)	6 (7.6%)	13 (13.4%)
HPV 18	6 (3.4%)	3 (3.8%)	3 (3.1%)
HPV 31	13 (7.4%)	3 (3.8%)	10 (10.3%)
HPV 33	5 (2.8%)	2 (2.5%)	3 (3.1%)
HPV 35	11 (6.2%)	5 (6.3%)	6 (6.2%)
HPV 39	16 (9.1%)	10 (12.7%)	6 (6.2%)
HPV 45	13 (7.4%)	4 (5.1%)	9 (9.3%)
HPV 51	8 (4.5%)	3 (3.8%)	5 (5.2%)
HPV 52	26 (14.8%)	18 (22.8%)	8 (8.2%)
HPV 53	6 (3.4%)	2 (2.5%)	4 (4.1%)
HPV 56	10 (5.7%)	4 (5.1%)	6 (6.2%)
HPV 58	10 (5.7%)	4 (5.1%)	6 (6.2%)
HPV 59	4 (2.3%)	1 (1.3%)	3 (3.1%)
HPV 6	2 (1.1%)	2 (2.5%)	0 (0.0%)
HPV 66	3 (1.7%)	2 (2.5%)	1 (1.0%)
HPV 67	17 (9.7%)	4 (5.1%)	13 (13.4%)
HPV 68	6 (3.4%)	5 (6.3%)	1 (1.0%)

### Factors associated with potential HR/HR HPV

In bivariate analysis (Table 4), the variables that showed an association with potential HR/HR HPV infection (P value ≤ 0.2) were age, level of education, marital status, and number of children. On multivariate regression, the independent significant factor associated with potential HR/HR HPV infection (P value ≤ 0.05) was age. Women between age 30–39 were the most likely to be infected with potential HR/HR HPV (AOR = 0.3, CI:0.2–0.7, *p* < 0.001) when compared to age 25–29(AOR = 0.3, CI:0.2–0.7, *p* = 0.002) and age 40+(AOR = 0.3; CI:0.1–0.6; *P* = 0.001). When age was adjusted for each variable in the age-adjusted model, marital status was the independent significant factor. Married women were more likely to be infected with potential HR/HR HPV than those who were single, divorced or widowed (AOR = 0.5; CI;0.3–0.9 *p* = 0.002).

**Table 4:** The results of bivariate, age-adjusted and multivariate analysis for sociodemographic and Reproductive health history and past sexual history

	Bivariable Logistic Regression		Age adjusted model		Multivariable Logistic Regression	
Variable	COR^1^	*p*-value	COR^1^	*p*-value	AOR^1^	*p*-value
Age						
16–24	Ref				Ref	
25–29	0.4 (0.2, 0.7)	**0.002**			0.3 (0.2, 0.7)	**0.002**
30–39	0.3 (0.2, 0.6)	**< 0.001**			0.3 (0.2, 0.6)	**< 0.001**
40+	0.3 (0.2, 0.6)	**< 0.001**			0.3 (0.1, 0.6)	**0.001**
**Site**						
Kisumu	Ref		Ref			
Siaya	1.3 (0.8, 2.1)	0.24	1.2 (0.7, 1.9)	0.54		
**Ever been married**						
No	Ref		Ref		Ref	
Yes	0.4 (0.3, 0.7)	**< 0.001**	0.5 (0.3, 0.9)	**0.020**	0.6 (0.3, 1.2)	0.13
**Type of visit**						
Initial screening	Ref		Ref			
Routine screening	0.6 (0.4, 0.9)	**0.021**	0.9 (0.5, 1.3)	0.51		
**Level of education**						
Primary/None	Ref		Ref		Ref	
Highschool	1.7 (1.1, 2.6)	**0.030**	1.3 (0.8, 2.2)	0.23	1.5 (0.9, 2.5)	0.12
College/University	1.5 (0.9, 2.6)	0.10	1.4 (0.8, 2.4)	0.21	1.4 (0.8, 2.6)	0.2
**Are you employed**						
No	Ref		Ref			
Yes	0.8 (0.5, 1.3)	0.35	1.1 (0.7, 1.8)	0.66		
**Number of children**						
0	Ref		Ref		Ref	
1	0.7 (0.3, 1.4)	0.30	0.6 (0.3, 1.3)	0.17	0.8 (0.3, 1.8)	0.5
2–3	0.5 (0.2, 0.9)	**0.031**	0.6 (0.3, 1.2)	0.13	0.9 (0.4, 2)	0.7
4+	0.4 (0.2, 0.9)	**0.027**	0.6 (0.3, 1.4)	0.25	1.1 (0.4, 2.8)	0.8
**Contraceptive use**						
No	Ref		Ref			
Yes	0.9 (0.6, 1.4)	0.68	1.1 (0.7, 1.7)	0.76		
**Had more than one sex partner**						
No	Ref		Ref		Ref	
Yes	1.4 (0.9, 2.3)	0.18	1.5 (0.9, 2.4)	0.13	1.3 (0.8, 2.3)	0.3
**Age at first sex**						
Below 15 years	Ref		Ref			
15–19 years	0.8 (0.5, 1.5)	0.53	0.7 (0.4, 1.4)	0.34		
Above 20 years	0.9 (0.5, 1.8)	0.82	0.9 (0.5, 1.9)	0.86		
**Had STI**						
No	Ref		Ref		Ref	
Yes	1.6 (1, 2.5)	0.055	1.7 (1.1, 2.8)	**0.029**	1.6 (1, 2.7)	0.056
**Had history of cervical cancer in the family**						
No	Ref		Ref			
Yes	0.9 (0.3, 2.2)	0.76	0.9 (0.4, 2.4)	0.86		
**Smokes cigarretes**						
No	Ref		Ref			
Yes	0.9 (0.2, 3.5)	0.85	1 (0.2, 4)	0.95		
**Ever used alcohal**						
No	Ref		Ref		Ref	
Yes	1.7 (1, 3.1)	0.065	1.7 (0.9, 3)	0.10	1.4 (0.7, 2.6)	0.3
**HIV status**						
Negative	Ref		Ref		Ref	
Positive	0.9 (0.6, 1.4)	0.65	1.4 (0.9, 2.2)	0.19	1.3 (0.8, 2.2)	0.3

^1^COR = Crude Odds Ratio, AOR = Adjusted Odds Ratio, CI = Confidence Interval

## Discussion

This study describes the potential HR/HR-HPV women attending reproductive health Clinics in Kisumu County (JOOTRH), an urban setting and Siaya County (Gobei Health Centre) a rural setting in Kenya. The two clinics support routine reproductive health services which include cervical cancer screening program. They are in western part of Kenya known to harbor a high burden of HIV. Of these women, potential HR/HR HPV prevalence of 36.3% is reported in this study. Past studies conducted in Kenya focus on the overall HPV-DNA prevalence (High risk and low risk) without specific emphasis on potential HR and HR HPV only. A study conducted at a reproductive health clinic in Nairobi revealed a HPV prevalence of 31.3% [20]. Similar studies conducted in Tigoni, Nairobi region reported a HPV prevalence of 32.7% [21]. Another study done among female sex workers in Kenya reported a baseline HPV prevalence of 23.6% [22]. Comparing the current and previous statistics, it is a clear indication that HPV and more specifically potential HR/HR HPV remains a common infection in LVB thus a potential indicator of possible rise in cervical cancer morbidity and mortality in the region. The prevalence of potential HR/HR HPV infection is higher compared to past national findings. This can be attributed to increased urbanization in the region and the endemic HIV burden. The prevalence of potential HR/HR-HPV genotypes among women attending reproductive health clinic in the rural setting (Gobei Health Center) and JOOTRH-Urban setting was 41.4% and 34.5% respectively. Past studies have reported varying trends of HPV prevalence in urban and rural areas [23, 24]. In a study conducted in Burundi, Urban areas reported a high prevalence of HR HPV associated with having multiple sex partners [24]. However, in this study, differences in prevalence could be attributed to the settings of the two facilities. JOOTRH- in the urban setting is a referral hospital that enjoys efficient services and resource coverage. By contrast, Gobei Health Center has limited resources and is in a rural setting. The two facilities attract varying numbers of women attending clinics hence the differences in sample sizes.

HPV 52, 67, 16, 31, 39, 45, 31 were the most prevalent potential HR/HR HPV types among women in this study. HPV 52 was highest at 14.8%. A systematic review and meta-analysis on genotype distribution of potential HR/HR HPV among women in Sub-Saharan Africa reveal HPV 16 and 52 as the main genotypes causing high-risk infection in Eastern and Southern Africa which includes Kenya [25].The prevalence of specific HPV types in the population does not have to equal the HPV types that cause cancer. However, the epidemiological knowledge of the specific types can be very interesting when evaluating the efficacy of vaccines and public health campains. Further, the high prevalence of other potential HR/HR HPVs such as HPV 67, 16, 31, 39 and 45 has been demonstrated previously in Sub-Saharan Africa and Kenya in particular [20, 22, 25–27]. HPV 52 and 16 were the most prevalent HR-HPV covered by the nonvalent vaccine. This study builds on the body of knowledge on the predominance of mixed potential HR/HR HPV types other than those in the HPV vaccines licensed and adopted in Kenya. For instance, the bivalent HPV vaccine (Cervarix) protects against HPV 16 and 18, the quadrivalent vaccine (Gardasil) protects against 6,11,16,18 and the Gardasil-9 against (6,11,16,18,31,33,45,52,58) This raises questions on the effectiveness of the current vaccines implemented in protection against HR HPV, particularly considering the HPV epidemiology in Kenya.

Further, the distribution of potential HR/HR HPV among HIV-positive and HIV-negative women in both reproductive health clinics was distinct. HPV 67, a potential HR HPV was noted as the most prevalent among HIV-positive patients. In a study done among Japanese women with cervical lesions, the genome analysis of HPV 67 suggested the carcinogenic potential of this rare genotype [28]. The high prevalence of HPV among HIV-positive patients indicates the need for further clinical and epidemiological follow-up study on the role of other HR and potential HR HPV in cervical diseases especially among immune-compromised population. Moreover, there is a need for development and adoption of commercial assays to make the detection of potential HR HPVs part of the HR HPV detection kits.

The bivariate analysis showed age, marital status, and parity as predictors of potential HR/HR HPV infection among women in this region (Table 4). However, on multivariate regression, only age remained as a significant risk factor. Infection with potential HR/HR HPV was highest among women between the age 30–39 years then decreased as the age of the women advanced to 40 + years. Most evaluations have established that the prevalence of HPV is highest in the younger age groups [29, 30]. Past research has shown that the first HPV infection is often acquired shortly after a woman becomes sexually active [31, 32]. HR HPV infection among younger women of less than 30 years often tends to be transient and screening in this age is likely to lead to detection of lesions that never progress to cervical cancer. However, as woman advances, the age-related HPV reduction has likely been attributed to a variety of factors including a decrease in involvement of sexual activity, clearance of HPV over a period and acquired immunity likely from previous infection. Supported by this evidence, it becomes necessary for Kenya to adhere to the WHO recommendations on the implementation of the two screenings in a woman’s lifetime, first by age 35 years and second by age 45 (WHO, 2020). Interestingly, HIV was not identified as a significant risk factor for HPV infection in this population both in bivariate and multivariate analysis despite being a HIV burdened region. It is critical to note that over the years, the Kenyan government have developed comprehensive Care Clinics for HIV patients in all major primary facilities which include JOOTRH and Gobei Health Center [33]. Nearly all identified HIV patients are subjected to routine care and follow-ups on treatment using appropriate Antiretroviral therapies. It is likely that the population of HIV positive women in this study form part of the program and are enrolled in Antiretroviral therapy at their respective clinics. A past epidemiological has shown that HIV patients on highly active Anti-Retroviral Therapy experience improved survival and reduction of new infection and development of precancerous lesions possible accounting for the findings [34].

Similarly, in the age-adjusted model of statistical analysis, being married was the only significant factor. The relationship status of women has often been considered a key determinant in HPV infection [35, 36]. The results of the current study are similar to the study findings in rural Uganda [37] where married women reported a high prevalence of HR HPV. However, being married has in the past drawn divergent results of HPV prevalence among women depending on the number of life partners that the woman or his partner has. Stable marital status, for instance, has often been viewed to protect an individual from HPV infection [38]. Nevertheless, this study did not consider the type of marriages of the women and the potential number of sex partners of their spouses. However, it is very common for men in Kenya both married and unmarried to have non-regular sex partners outside their monogamous setting [27].

### Strength and limitations

This is the first study in the region to report extended genotyping data for both potential HR and HR HPV among women in rural and Urban settings in the post-pandemic era in LVB. Our findings add to the epidemiological knowledge on burden, genotypic distribution and potential factors associated with potential HR/HR HPV infection and disease progression among women in LVB.

The main limitation of this study is the source of study participants from facilities only. Given the fact that JOOTRH is a Referral hospital (level 5), it offers more specialized services and resources than health centers (level 3). This leads to discrepancies in cervical screening uptake, diagnoses treatment options, and outcomes, hence impacting the study results.

Further, the urban and rural populations differ in demographics, health needs, and access to healthcare. This can lead to significant differences in the patient populations at the two health facilities, potentially causing bias in the study results.

It is necessary to obtain more precise data on prevalence rates of potential HR/HR HPV in the entire Lake Victoria Region.

## Conclusion

Our findings reveal that potential HR/HR HPV infection remains common in the LVR region when compared to the global prevalence. The main circulating HPV genotypes are 52, 67, 16, 31, 39, 45 among women in rural and urban settings of the selected reproductive health clinics. Further, a follow-up study will focus on the detailed analysis of HPV quantification of the viral load in the context of HIV infections in a subsequent publication. The main factor associated with increased risk of potential HR/HRHPV was shown in this study to be age, particularly with women aged between 30 and 39 to present higher prevalence of HPV infection. Given these results, the ongoing HPV national vaccination coverage is critical and should be undertaken before onset of sexual activities among the young women. The dominance of mixed HPV genotypes supports the need for more comprehensive vaccine in Kenya, accounting for the most prevalent HR-HPV types in the region. Self-evidently, it is urgent that Kenya health entities promote Gardsail-9, the nonavalent vaccine, covering both LR and most of the HR HPV types known to cause pre- cancerous lesions, in order to control and decrease HPV infection among women in the region.

### Electronic supplementary material

Below is the link to the electronic supplementary material.

Supplementary Material 1

## Data Availability

The datasets generated and/or analysed during the current study are not publicly available due to the limitations of ethical approval involving the patient data and anonymity but are available from the corresponding author upon reasonable requests.

## Abbreviations

HPV: Human Papillomavirus
HR-HPV: High Risk Human Papillomavirus
HIV: Human Immunodeficiency Virus
LR: Low risk human papillomavirus
LVB: Lake Victoria Basin
JOOTRH: Jaramogi Oginga Odinga Teaching and Referral Hospital
