# Supplementary material for: Prevalence and genotype distribution of potential high-risk and high-risk human papillomavirus among women attending selected reproductive health clinics in lake victoria basin-kenya: a cross-sectional study
Source: BMC Womens Health. 2024 Aug 24;24:468. doi: 10.1186/s12905-024-03303-9 (PMC11344458; doi:10.1186/s12905-024-03303-9)
Supplement: Supplementary file 1 — Supplementary Material 1 [file 12905_2024_3303_MOESM1_ESM.docx]

**Supplementary Information**

**Questionnaire**

Facility: ……………………………….

Participant Code……………………….

**Socio-Demographics**

1. How old are you?................
2. What is your marital status?
3. Married
4. Single
5. Widowed
6. Divorced/Separated
7. How many Children do you have?...........................
8. What do you do for a living?
9. Employed
10. Self-employed/Business
11. Unemployed
12. What is your level of education?
13. Unable to read/Write
14. Primary
15. High School
16. College/University

**Previous Sexual/Reproductive History**

1. In the past 6 months, have you been on contraceptives?
2. Yes
3. No
4. If Yes, which type of contraceptives have you been using?
5. Oral Contraceptives
6. IUCD
7. Implant
8. Depo
9. Tubal Ligation
10. Condom
11. Other, Specify……………….
12. At what age did you first have sex?
13. Below 15 years
14. 15-20 years
15. 20-25 years
16. Above 25 years
17. Have you ever had more than one sexual partner in the past?
18. Yes
19. No
20. Have you ever had any sexually transmitted diseases?
21. Yes
22. No
23. Has anyone in your family ever had cervical cancer?
24. Yes
25. No
26. Do you smoke cigarettes
27. Yes
28. No
29. Do you use alcohol?
30. Usually
31. Occasionally
32. Never
33. Regarding cervical cancer screening, please choose between the two
34. Initial Screening-This is the first time you want to be screened
35. Routine screening- This is part of your routine healthcare practice
36. HIV Sero-status (To be ticked by research assistant upon confirmation from MOH HIV surveillance registry)
37. HIV positive
38. HIV Negative
